# The carotid body in sepsis: From homeostatic defense to immunometabolic dysregulation

**DOI:** 10.1016/j.bbih.2026.101325

**Published:** 2026-08-12

**Authors:** Natalia C.S. Branco, Isabela S. Ferracioli, Evelin C. Cárnio

**Affiliations:** a Medical School of São José do Rio Preto, FAMERP, São José do Rio Preto, SP, Brazil; b Medical School of Ribeirão Preto, University of São Paulo, Ribeirão Preto, São Paulo, Brazil; c Nursing School of Ribeirão Preto, University of São Paulo, Ribeirão Preto, São Paulo, 14040-902, Brazil

**Keywords:** Sepsis, Carotid body, Autonomic nervous system, Lactate, Hypothermia, Bioenergetic failure

## Abstract

Sepsis is a life-threatening syndrome caused by a dysregulated host response to infection and involves failure of immune, metabolic, respiratory, cardiovascular, thermal, endocrine, and autonomic regulation. In this review, we propose that the carotid body may act as a peripheral interoceptive checkpoint in the transition from adaptive host defense to immunometabolic decompensation. The discussion is organized around four interconnected dimensions. First, we examine inflammatory and metabolic signals that may be detected by the carotid body during sepsis, including cytokines, lactate, glucose, hypoxemia, acid-base disturbances, and other energetic stress signals. Second, we discuss the respiratory consequences of carotid body activation or dysfunction, with emphasis on ventilation, hypoxic chemosensitivity, and ventilatory responses during systemic inflammation. Third, we address cardiovascular and autonomic consequences, including sympathetic activation, vascular tone, arterial pressure, organ perfusion, and recruitment of the splanchnic anti-inflammatory pathway. Fourth, we consider inflammation-induced carotid body remodeling and the resulting loss of sensory fidelity. Experimental evidence indicates that circulating TNF-α activates carotid body afferents and recruits sympathetic anti-inflammatory pathways, whereas severe or persistent inflammation can alter carotid body structure, chemosensory gain, and afferent output. Although direct clinical evidence remains limited, this framework identifies the carotid body as a testable neuroimmune interface. Early stimulus-coupled activation may support respiratory, cardiovascular, autonomic, metabolic, and immune adaptation, whereas persistent inflammation may produce elevated tonic afferent activity, reduced stimulus-evoked responsiveness, and impaired sensory discrimination.

## Highlights

• Carotid body may link immune–metabolic signals to autonomic control.
• Septic hypothermia may reflect metabolic restraint or homeostatic failure.
• Inflammation-induced carotid body remodeling may impair sensory fidelity.

## 1 Introduction: sepsis as a failure of coordinated homeostatic regulation

Infection challenges the organism with a dual requirement: the host must generate an inflammatory response strong enough to control pathogens while simultaneously activating regulatory mechanisms that prevent host defense from becoming self-injurious. Survival therefore depends on coordinated local, endocrine, and neural pathways that limit excessive inflammation while preserving antimicrobial defense (Kotas and Medzhitov, 2015; Nathan, 2002). Locally, immune, endothelial, and stromal cells regulate the magnitude and duration of inflammation through cytokine control, macrophage reprogramming, efferocytosis, specialized pro-resolving lipid mediators, and tissue repair mechanisms (Nathan, 2002; Medzhitov, 2008; Buckley et al., 2014; Basil and Levy, 2016). When inflammation becomes systemic, endocrine regulation is recruited, particularly through the hypothalamic-pituitary-adrenal axis, which promotes glucocorticoid release to restrain cytokine production, modulate leukocyte activation, and support metabolic and cardiovascular adaptation (Chrousos, 1995; Bellavance and Rivest, 2014; Vandewalle and Libert, 2020).

Systemic inflammation is also controlled by autonomic reflex circuits. The inflammatory reflex detects peripheral inflammatory signals through afferent sensory pathways and recruits efferent autonomic outputs that modulate cytokine production, immune organ activity, energy metabolism, and cardiovascular stability (Tracey, 2002; Pavlov and Tracey, 2012; Martelli et al., 2014). Although this reflex was initially associated mainly with vagal cholinergic signaling, subsequent evidence indicates that sympathetic circuits, particularly the splanchnic sympathetic pathway, are central to the control of systemic inflammation during severe inflammatory challenges (Borovikova et al., 2000; Pavlov and Tracey, 2012; Wang et al., 2003; Rosas-Ballina et al., 2011; Martelli et al. 2014, 2019; Martelli et al., 2016; Komegae et al., 2018; McKinley et al., 2022). These neural pathways provide a physiological basis for considering peripheral sensory organs as potential detectors of circulating inflammatory and metabolic information.

When local, endocrine, and neural regulatory mechanisms fail, infection can progress to sepsis, defined as life-threatening organ dysfunction caused by a dysregulated host response to infection (Singer et al., 2016). Sepsis is therefore not simply a state of excessive inflammation, but a failure of coordinated homeostatic regulation involving immune, neuroendocrine, metabolic, endothelial, thermal, respiratory, cardiovascular, and autonomic dysfunction (Singer et al., 2016; Wiersinga and van der Poll, 2022; Cárnio et al., 2025). Hyperinflammation and immunosuppression may coexist across time, tissues, and patients rather than occurring as separate linear phases (Wiersinga and van der Poll, 2022; van der Poll et al., 2017; Hotchkiss et al., 2013; Hotchkiss et al., 2016). Septic immunosuppression is characterized by T-cell apoptosis and exhaustion, increased immune checkpoint expression, impaired antigen presentation, reduced monocyte HLA-DR, expansion of regulatory T cells and myeloid-derived suppressor cells, increased anti-inflammatory signaling, and susceptibility to secondary infections (Hotchkiss et al., 2013; Gao et al., 2025; Liu et al., 2022; Zhang et al., 2023). Importantly, this state may reflect not only passive immune failure but also a maladaptive extension of compensatory mechanisms designed to restrain inflammation (Hotchkiss et al., 2013; Hotchkiss et al., 2016; Wiersinga and van der Poll, 2022; Gao et al., 2025).

Sepsis is also a profound metabolic and thermoregulatory disorder. Immune cells and organs undergo major shifts in substrate use, glycolytic activity, mitochondrial function, lactate production, and ATP availability. These changes may initially redistribute energy toward fever, immune activation, cardiovascular compensation, tissue repair, and pathogen control, but can become maladaptive when inflammation persists, leading to lactate accumulation, mitochondrial injury, ATP depletion, impaired cellular repair, and progressive organ dysfunction (Brealey and Singer, 2003; Singer, 2014; Protti and Singer, 2006; Wiersinga and van der Poll, 2022). Body temperature provides an integrated readout of this immunometabolic state. Fever and hypothermia are associated with distinct patterns of metabolic demand, disease severity, and outcome, and may represent either coordinated adaptive responses or different stages of homeostatic failure (Evans et al., 2015; Romanovsky and Székely, 1998; Garami et al., 2018; Kushimoto et al., 2013; Rumbus et al., 2017; Thomas-Rüddel et al., 2021).

The transition from coordinated host defense to respiratory, cardiovascular, autonomic, and metabolic decompensation requires sensory checkpoints capable of detecting changes in the internal milieu. The carotid bodies are small paired organs located at the carotid bifurcations and are strategically positioned to monitor blood before it reaches the brain. Classically, they are the principal peripheral chemoreceptors for reductions in arterial O_2_, increases in CO_2_, and changes in pH. More recent evidence indicates that they can also respond to selected inflammatory and metabolic signals, including cytokines, lactate, and glucose (Kumar and Prabhakar, 2012; Iturriaga, 2018; Iturriaga et al., 2022; Gao et al., 2014; Joyner et al., 2018; Torres-Torrelo et al., 2021; Katayama et al., 2022a, Katayama et al., 2022b). The following sections therefore examine the carotid body in sepsis according to the signals it may detect, the respiratory and cardiovascular/autonomic consequences of its activation, and the possibility that inflammation remodels the sensor itself.

## 2 Inflammatory and metabolic signals detected by the carotid body during sepsis

### 2.1 Hypoxemia, carbon dioxide, and acid-base disturbances

The best-established function of the carotid body is the detection of arterial hypoxemia, hypercapnia, and changes in pH. During hypoxemia, glomus cells depolarize and release neurotransmitters, including ATP, which acts locally to activate afferent fibers of the carotid sinus nerve. These fibers project primarily to the nucleus of the solitary tract in the brainstem, where chemosensory information is integrated with other visceral inputs and translated into respiratory, cardiovascular, and autonomic responses (Kumar and Prabhakar, 2012; Iturriaga, 2018; Iturriaga et al., 2022). This classical function is directly relevant to sepsis because arterial hypoxemia, acidemia, and alterations in arterial CO_2_ levels may coexist during respiratory and circulatory dysfunction. Ventilation–perfusion mismatch, respiratory failure, impaired oxygen delivery, and tissue hypoperfusion are not themselves direct carotid body stimuli, but they may indirectly influence carotid body activity by altering arterial blood gases, pH, or circulating metabolites. The carotid body is therefore positioned to detect the arterial consequences of respiratory and circulatory disturbances associated with systemic infection and organ dysfunction. ATP should be considered separately at the levels of local neurotransmission and cellular bioenergetics. Within the carotid body, ATP is released by activated glomus cells and acts as an important extracellular transmitter in chemosensory communication with carotid sinus nerve endings (Zapata et al., 2011; Conde et al., 2018). This local neurotransmitter function is distinct from changes in intracellular ATP availability caused by mitochondrial dysfunction. In sepsis, impaired mitochondrial oxygen utilization and ATP production can occur in multiple tissues and may persist despite improvement in macrocirculatory variables, contributing to cellular dysfunction and organ failure (Brealey and Singer, 2003; Protti and Singer, 2006; Singer, 2014). These systemic bioenergetic abnormalities should not be interpreted as circulating signals directly sampled by the carotid body. Whether sepsis-related mitochondrial dysfunction also develops within carotid body cells and consequently alters ATP synthesis, ATP release, or purinergic neurotransmission has not been established. ATP should therefore be regarded primarily as a locally released mediator of carotid body chemosensory transmission, whereas the potential effects of intrinsic carotid body bioenergetic dysfunction during sepsis remain an important but unresolved question.

### 2.2 Inflammatory cytokines and infection-related signals

The carotid body is increasingly recognized as an immunosensory organ rather than a sensor restricted to blood gases. Glomus cells and associated sensory pathways express receptors for inflammatory mediators, including TNF-α, IL-1β, and IL-6, and experimental studies show that these signals can modify carotid sinus nerve activity and activate brainstem pathways involved in autonomic regulation (Wang et al., 2002; Shu et al., 2007; Fan et al., 2009; Zapata et al., 2011; Iturriaga et al., 2022). Katayama et al. provided direct evidence that circulating TNF-α is detected by the carotid body and that this afferent signal is conveyed through the nucleus of the solitary tract and rostral ventrolateral medulla to recruit a splanchnic sympathetic anti-inflammatory response (Katayama et al., 2022a, Katayama et al., 2022b). More recently, carotid body sensing of systemic inflammation was shown to contribute to recruitment of the splanchnic anti-inflammatory pathway (Rosin et al., 2026).

This evidence is especially relevant in sepsis, in which circulating cytokines are accompanied by pathogen-associated molecular patterns, endothelial activation, metabolic stress, and altered tissue perfusion. The carotid body and its associated sensory ganglia also express Toll-like receptor 4, the canonical receptor for lipopolysaccharide, supporting the possibility that infection-related signals can act directly on the carotid chemoreceptor pathway rather than only through secondary changes in oxygenation, pH, or circulation (Fernández et al., 2011). Thus, the carotid body may detect inflammation through at least two related mechanisms: sensing circulating cytokines as physiological information and responding directly to pathogen-associated inflammatory signaling. The distinction is important because transient cytokine sensing may recruit protective reflexes, whereas intense or prolonged inflammatory signaling may modify the sensor itself, as discussed in Section 5.

### 2.3 Lactate and other energetic stress signals

Lactate is one of the most widely used metabolic markers in sepsis. Elevated serum lactate is associated with disease severity, tissue hypoperfusion, impaired oxygen utilization, and increased mortality risk, and current guidelines recommend using lactate to guide resuscitation in patients with elevated levels while interpreting it in the broader clinical context (Evans et al., 2021; Garcia-Alvarez et al., 2014; Hernandez et al., 2019). Hyperlactatemia is not simply a direct measurement of oxygen debt. It can result from accelerated aerobic glycolysis, adrenergic stimulation, impaired pyruvate dehydrogenase activity, mitochondrial dysfunction, reduced hepatic clearance, microcirculatory alterations, and true tissue hypoxia (Garcia-Alvarez et al., 2014; Hernandez et al., 2019; Evans et al., 2021). Lactate is therefore an integrated marker of cellular stress, altered metabolism, circulatory dysfunction, and impaired clearance.

Lactate is also a biologically active molecule. It can influence macrophage polarization, cytokine production, T-cell and dendritic-cell function, and the balance between inflammatory and anti-inflammatory responses. These effects may involve lactate transporters, receptor-mediated signaling, and epigenetic mechanisms such as histone lactylation, which links metabolic state to gene transcription and immune adaptation (Zhang et al. 2019, 2024; Liu et al., 2024). This broader interpretation is relevant to the carotid body because lactate may provide the nervous system with information about energetic imbalance rather than functioning only as a terminal metabolic byproduct.

Torres-Torrelo et al. demonstrated that carotid body glomus cells can function as lactate sensors. In their model, lactate enters glomus cells, alters the cytosolic NADH/NAD^+^ ratio, activates cation channels, depolarizes the cells, and promotes action-potential firing and calcium influx. Lactate and hypoxia appear to use distinct initial sensing mechanisms but converge on final pathways that activate glomus cells and potentiate compensatory cardiorespiratory reflexes (Torres-Torrelo et al., 2021). Although this mechanism has not been directly demonstrated in sepsis, it provides a plausible route through which hyperlactatemia could influence respiratory and cardiovascular/autonomic responses. In parallel, impaired mitochondrial function and reduced ATP availability could modify purinergic transmission and thereby distort the sensory representation of energetic stress (Brealey and Singer, 2003; Singer, 2014; Garcia-Alvarez et al., 2014; Hernandez et al., 2019; Wiersinga and van der Poll, 2022; Zapata et al., 2011).

### 2.4 Glucose availability and the thermal-metabolic context

Some studies suggest that carotid body glomus cells can respond to low glucose and participate in counter-regulatory responses to hypoglycemia, including sympathetic and endocrine activation (Gao et al., 2014; Conde et al., 2018; Joyner et al., 2018). These findings indicate that the carotid body can link circulating substrate availability to autonomic and hormonal outputs. Sepsis may involve hyperglycemia, altered glucose utilization, later hypoglycemia, increased glycolysis, and disturbed hepatic and peripheral metabolism. Direct evidence that carotid body glucose sensing shapes the septic response is lacking, but changes in glucose availability may contribute to the combined metabolic information reaching the carotid body.

Body temperature should be interpreted differently: it is a well-established integrated output of immune, neuroendocrine, metabolic, cardiovascular, and autonomic regulation, but evidence for direct temperature sensing by the carotid body during sepsis is less developed. Fever is a regulated acute-phase response that can enhance immune-cell activity and antimicrobial defense but also increases oxygen consumption, substrate mobilization, thermogenesis, and cardiovascular work (Evans et al., 2015; Wrotek et al., 2021; Garami et al., 2018). Spontaneous hypothermia is generally associated with greater organ dysfunction and mortality, although it may in some settings represent an energy-conserving or tolerance-oriented response when the metabolic cost of fever becomes unsustainable (Kushimoto et al., 2013; Rumbus et al., 2017; Thomas-Rüddel et al., 2021; Romanovsky and Székely, 1998; Garami et al., 2018; Ganeshan et al., 2019; Fan and Gautron, 2026). Thermal conditions can modify carotid body chemosensitivity, and heat-induced hyperventilation has been linked to carotid body hyperexcitability in humans (Kumar and Prabhakar, 2012; Joyner et al., 2018; Gibbons et al., 2022). Nevertheless, temperature is best treated here as a modulator and clinical readout of the immunometabolic state rather than as a confirmed sepsis-related carotid body ligand.

Taken together, arterial blood during sepsis carries a complex combination of hypoxemia, changes in CO_2_ and pH, cytokines, pathogen-associated signals, lactate, changes in glucose availability, and energetic stress. The carotid body may therefore operate as a polymodal interoceptive node that samples these convergent signals and transmits an integrated afferent representation to the brainstem. The physiological meaning of this input depends not only on what the carotid body detects but also on how the central nervous system translates the signal into respiratory and cardiovascular/autonomic outputs. The principal effects of sepsis-related inflammatory and metabolic factors on carotid body structure and function are summarized in Table 1.

**Table 1:** Effects of sepsis-related inflammatory and metabolic factors on carotid body structure and function.

Factor	Carotid body target/mechanism	Observed effect	Potential consequence in sepsis	Evidence limitation
TNF-α	TNF receptors on glomus cells and carotid body afferents; signaling is transmitted through the NTS and RVLM to the splanchnic sympathetic nerves.	TNF-α increases carotid body afferent discharge and splanchnic sympathetic nerve activity. Carotid body ablation or splanchnic denervation increases plasma and splenic cytokine responses; TNF-α may also modify hypoxic chemosensitivity (Katayama et al., 2022a, Katayama et al., 2022b; Fernández et al., 2008, 2011; Iturriaga et al., 2022).	May recruit an adaptive anti-inflammatory reflex and coordinate sympathetic cardiovascular responses. Persistent exposure could alter sensory gain and contribute to maladaptive autonomic or immune regulation.	Evidence is mainly from acute exogenous TNF-α challenges and animal preparations; infection-driven human sepsis has not been studied directly.
IL-1β	IL-1 receptor type I on glomus cells; inhibition of outward K^+^ currents increases membrane excitability.	IL-1β increases intracellular Ca^2+^ and carotid sinus nerve firing (Wang et al., 2002; Shu et al., 2007; Iturriaga et al., 2022).	May increase chemosensory gain and respiratory or autonomic drive. Sustained exposure could saturate afferent output and reduce discrimination among hypoxia, acidosis, and metabolic signals.	Predominantly rodent cellular and tissue evidence; no direct demonstration of its contribution to cardiorespiratory dysfunction in sepsis.
IL-6	Cytokine signaling in glomus cells, involving Ca^2+^-dependent catecholamine secretion.	IL-6 increases intracellular Ca^2+^ and catecholamine release from rat carotid body glomus cells (Fan et al., 2009).	Could modify neurotransmitter release, chemosensory gain, ventilation, and autonomic output during systemic inflammation.	Evidence is based mainly on rat glomus-cell preparations; effects during endotoxemia, infection-based sepsis, and human disease remain unknown.
LPS and TLR4-related signaling	TLR4/MyD88-dependent NF-κB, p38 MAPK, and ERK signaling in carotid body cells and associated sensory ganglia; inflammatory remodeling of glomic tissue.	LPS increases basal chemosensory discharge, activates inflammatory pathways, causes disorganization of glomic lobules and inflammatory-cell accumulation, and reduces responses to hypoxia and nicotine. Carotid body ablation also blunts RVLM and splanchnic activation during endotoxemia (Fernández et al., 2008, 2011; Rosin et al., 2026).	May initially recruit the splanchnic anti-inflammatory pathway, but sustained signaling may cause glomitis, reduce chemosensory dynamic range, and impair ventilatory and autonomic compensation.	Findings derive from acute, frequently high-dose endotoxemia in anesthetized animals; LPS models do not reproduce the full complexity of infection-driven human sepsis.
Lactate	Uptake into glomus cells increases the cytosolic NADH/NAD^+^ ratio, activates cation channels, depolarizes the cells, and promotes Ca^2+^ influx.	Lactate activates glomus cells and potentiates compensatory cardiorespiratory reflexes (Torres-Torrelo et al., 2021).	Sepsis-associated hyperlactatemia may augment or distort ventilation, sympathetic activation, arterial pressure, and perfusion responses.	The mechanism has not been tested during endotoxemia or sepsis; acute lactate exposure in experimental preparations may not represent the metabolic complexity of septic patients.
Hypoxemia, hypercapnia, and acidemia	Classical O_2_-, CO_2_-, and pH-sensitive mechanisms in glomus cells; depolarization leads to ATP release and carotid sinus nerve activation.	Activation increases ventilation and recruits sympathetic cardiovascular responses that support gas exchange, acid-base balance, arterial pressure, and oxygen delivery (Kumar and Prabhakar, 2012; Iturriaga, 2018; Iturriaga et al., 2022).	Provides compensatory respiratory and circulatory responses during sepsis. Reduced chemosensitivity could worsen gas exchange, acid-base disturbances, hypotension, and organ hypoperfusion.	The physiology is well established, but the specific contribution of carotid body dysfunction is difficult to distinguish from pulmonary, central nervous system, and circulatory abnormalities in sepsis.
Glucose availability	Proposed low-glucose sensing by glomus cells and activation of sympathetic and endocrine counter-regulatory pathways.	Some studies report glomus-cell activation by low glucose and carotid body participation in counter-regulatory responses to hypoglycemia (Gao et al., 2014; Conde et al., 2018; Joyner et al., 2018).	May link sepsis-associated dysglycemia to autonomic, endocrine, and metabolic adaptation.	Direct low-glucose sensing remains controversial and preparation-dependent and has not been demonstrated during sepsis.
Temperature and thermal stress	Thermal state appears to modulate tonic carotid body activity and hypoxic sensitivity rather than acting as a confirmed sepsis-related ligand.	Heat strain increases tonic carotid body activity and hypoxic sensitivity, and transient hyperoxia reduces the additional ventilatory drive during exercise heat stress (Gibbons et al., 2022).	Could modify respiratory drive and cardiorespiratory workload during fever or hypothermia and shape the context in which other arterial signals are encoded.	Evidence comes from healthy humans exposed to exercise heat stress; direct temperature sensing and relevance to acute sepsis remain unproven.
ATP and local purinergic signaling	ATP released from activated glomus cells acts on purinergic receptors at carotid sinus nerve endings.	Extracellular ATP provides an important excitatory signal for carotid body chemosensory neurotransmission (Zapata et al., 2011; Conde et al., 2018).	Intrinsic bioenergetic dysfunction within the carotid body could impair ATP synthesis, release, or afferent transmission and thereby contribute to loss of sensory fidelity.	Systemic ATP depletion is not a circulating stimulus directly sampled by the carotid body, and no study has established that sepsis alters local ATP-dependent transmission.
Persistent inflammatory exposure and remodeling	Glomic lobules, type II cells, dopamine content, connective tissue, inflammatory-cell recruitment, and afferent chemosensory pathways.	Inflammatory exposure causes structural disorganization, connective-tissue expansion, inflammatory-cell accumulation, reduced type II cell volume, increased dopamine content, attenuated hypoxic sensitivity, and impaired ventilatory responses (Fernández et al., 2008, 2011; Master et al., 2016).	May reduce stimulus discrimination, with elevated tonic afferent activity but attenuated stimulus-evoked responses, thereby impairing respiratory, cardiovascular, autonomic, metabolic, and immune regulation during septic decompensation.	Evidence is derived from endotoxemia and developmental animal models; no causal evidence is available in adult infection-based sepsis or humans.

Abbreviations: ATP, adenosine triphosphate; ERK, extracellular signal-regulated kinase; IL, interleukin; LPS, lipopolysaccharide; MAPK, mitogen-activated protein kinase; MyD88, myeloid differentiation primary response 88; NADH/NAD^+^, reduced/oxidized nicotinamide adenine dinucleotide; NF-κB, nuclear factor kappa B; NTS, nucleus of the solitary tract; RVLM, rostral ventrolateral medulla; TLR4, Toll-like receptor 4; TNF-α, tumor necrosis factor alpha.

## 3 Respiratory consequences of carotid body activation or dysfunction

### 3.1 Ventilatory responses to carotid body activation

The respiratory effects of carotid body activation are best established in response to hypoxemia. Glomus-cell depolarization and transmitter release activate the carotid sinus nerve, and afferent input to the nucleus of the solitary tract increases ventilation to restore arterial oxygenation and acid-base balance (Kumar and Prabhakar, 2012; Iturriaga, 2018; Iturriaga et al., 2022). In sepsis, this reflex may be recruited by hypoxemia, acidosis, hypercapnia, lactate, inflammatory mediators, or combinations of these stimuli. Because lactate and hypoxia converge on glomus-cell activation, lactate sensing could reinforce cardiorespiratory compensation even when hyperlactatemia is not caused exclusively by tissue hypoxia (Torres-Torrelo et al., 2021).

The respiratory response should therefore be viewed as the product of integrated arterial chemistry rather than as a reaction to oxygen alone. In early or controlled systemic inflammation, increased carotid body activity may support ventilation and help maintain gas exchange and pH. This response could be adaptive when it matches metabolic demand and lung function. However, excessive sensory gain could increase respiratory drive and workload, whereas inadequate or distorted signaling could blunt compensatory ventilation. The functional direction is likely to depend on the intensity and duration of inflammation, the balance among competing stimuli, and whether the carotid body remains structurally and functionally intact.

### 3.2 Changes in hypoxic chemosensitivity during systemic inflammation

Studies of experimental endotoxemia demonstrate that systemic inflammation can alter both basal carotid body discharge and stimulus-evoked respiratory responses. In cats, intravenous lipopolysaccharide produced systemic manifestations compatible with septic shock and increased basal carotid chemosensory discharge, yet reduced ventilatory and chemosensory responses to hypoxia and nicotine (Fernández et al. 2008, 2011). This combination is physiologically important: a sensor may become tonically active while simultaneously losing the capacity to encode additional changes in arterial oxygenation. Such a reduction in dynamic range would impair the fidelity of hypoxic signaling even when baseline afferent activity is elevated.

Inflammatory cytokines can also alter glomus-cell excitability and neurotransmitter release. IL-1β inhibits outward potassium currents, increases intracellular Ca^2+^, and increases carotid sinus nerve firing, while IL-6 increases intracellular Ca^2+^ and catecholamine secretion in rat glomus cells. TNF-α and its receptors are present in the carotid body, and TNF-α can modulate chemosensory activity, particularly under hypoxic conditions (Wang et al., 2002; Shu et al., 2007; Fan et al., 2009; Fernández et al. 2008, 2011; Zapata et al., 2011; Iturriaga et al., 2022). These findings suggest that the ventilatory response during sepsis may be altered not only by pulmonary or central nervous system dysfunction but also by inflammatory modification of the peripheral chemoreceptor pathway.

### 3.3 Respiratory consequences of impaired sensory fidelity

If carotid body signaling becomes distorted, elevated tonic afferent activity may coexist with reduced stimulus-evoked responsiveness and impaired stimulus discrimination. Reduced hypoxic chemosensitivity could limit ventilatory responses when oxygen tension falls, whereas persistent basal activation could contribute to disproportionate respiratory drive. Inflammation-related changes may also interfere with the integration of hypoxia, acidosis, lactate, glucose, and thermal context, making the afferent output less representative of the actual physiological need. The result could be impaired matching between ventilation, metabolic demand, oxygen delivery, and acid-base status.

Body temperature may further influence this respiratory dimension. Fever increases metabolic rate and oxygen demand and can increase ventilatory requirements, whereas hypothermia reduces metabolic demand but may accompany advanced circulatory, endocrine, mitochondrial, or autonomic failure (Garami et al., 2018; Ganeshan et al., 2019; Wrotek et al., 2021; Singer, 2014). Experimental evidence that thermal conditions alter carotid body chemosensitivity and that carotid body hyperexcitability contributes to heat-induced hyperventilation supports an interaction between thermal state and peripheral chemoreflex control (Joyner et al., 2018; Gibbons et al., 2022). Direct evidence in septic patients is not available, and temperature should not be interpreted as proof of carotid body dysfunction. Rather, it may define the metabolic context in which respiratory chemoreflexes operate.

The clinical relevance of altered carotid chemoreflexes in infection-related disease is supported indirectly by emerging human data showing abnormal carotid body sensitivity and ventilatory responses in post-infectious syndromes (El-Medany et al., 2024). These findings do not establish the same mechanism in acute sepsis, but they support the translational feasibility of evaluating carotid chemoreflex sensitivity in patients. Future studies should determine whether changes in hypoxic ventilatory responsiveness occur across septic phenotypes and whether they correlate with lactate, temperature, organ dysfunction, respiratory support requirements, and outcome.

## 4 Cardiovascular and autonomic consequences of carotid body activation or dysfunction

### 4.1 Brainstem integration and autonomic recruitment

Carotid sinus nerve afferents project primarily to the nucleus of the solitary tract, which connects with the rostral ventrolateral medulla, dorsal motor nucleus of the vagus, nucleus ambiguus, and other autonomic regions. Through these circuits, carotid body input can influence both sympathetic and parasympathetic outputs (Kumar and Prabhakar, 2012; Iturriaga, 2018; Iturriaga et al., 2022; Katayama et al., 2022). The carotid body also receives autonomic innervation that may regulate local blood flow, glomus-cell activity, and chemoreflex sensitivity. Its output is therefore shaped by blood-borne stimuli and by autonomic modulation of sensory gain. Even in its classical role, the carotid body is not merely a respiratory sensor; it links arterial chemistry to coordinated autonomic adaptation.

This organization is particularly relevant to sepsis because respiratory, cardiovascular, endocrine, metabolic, thermal, and immune responses must be coordinated rather than activated independently. Afferent information about hypoxemia, acidosis, lactate, glucose availability, and cytokines can be integrated with other visceral and central signals, after which autonomic pathways may adjust vascular tone, heart rate, adrenal activity, hepatic glucose production, thermogenesis, splenic immune function, and organ perfusion. The carotid body may therefore function as an upstream component of a broader homeostatic network rather than as the sole controller of any individual response.

### 4.2 Sympathetic activation, vascular tone, arterial pressure, and organ perfusion

Carotid body activation contributes to increases in sympathetic nerve activity and to adjustments in arterial pressure, heart rate, and vascular resistance (Kumar and Prabhakar, 2012; Iturriaga, 2018; Iturriaga et al., 2022). During sepsis, these responses could support perfusion when arterial pressure or oxygen delivery is threatened. Sympathetic activation may redistribute blood flow, maintain arterial pressure, mobilize metabolic substrates, and support adrenal responses. These effects may initially be protective, particularly when they are proportional to the severity of the arterial disturbance.

The same pathway may become maladaptive when afferent signaling or central integration is excessive, persistent, or distorted. Excessive sympathetic activity can increase cardiovascular workload and metabolic demand, whereas inadequate activation may fail to sustain vascular tone or organ perfusion. Because hyperlactatemia may reflect adrenergic stimulation, microcirculatory dysfunction, altered mitochondrial metabolism, impaired clearance, or tissue hypoxia, carotid body sensing of lactate could participate in a feedback loop connecting metabolic stress to sympathetic and cardiovascular compensation (Garcia-Alvarez et al., 2014; Hernandez et al., 2019; Torres-Torrelo et al., 2021). This remains a mechanistic hypothesis rather than a demonstrated pathway in human sepsis.

### 4.3 Recruitment of the splanchnic anti-inflammatory pathway

The strongest direct evidence linking the carotid body to autonomic immune control comes from the response to circulating TNF-α. Katayama et al. demonstrated that TNF-α activates carotid body afferents, recruits neurons in the nucleus of the solitary tract that project to the rostral ventrolateral medulla, increases splanchnic sympathetic nerve activity, and limits cytokine concentrations in plasma and spleen. Carotid body removal or splanchnic sympathetic denervation intensified cytokine responses, supporting the existence of a carotid body-brainstem-splanchnic sympathetic anti-inflammatory reflex (Katayama et al., 2022a, Katayama et al., 2022b). Rosin et al. subsequently showed that carotid body sensing of systemic inflammation contributes to recruitment of the splanchnic anti-inflammatory pathway (Rosin et al., 2026).

These findings extend the broader inflammatory-reflex literature, in which autonomic pathways regulate cytokine production and immune organ activity (Tracey, 2002; Pavlov and Tracey, 2012). Although early models emphasized vagal cholinergic signaling (Borovikova et al., 2000; Wang et al., 2003; Rosas-Ballina et al., 2011), experimental work indicates that the splanchnic sympathetic pathway is a major efferent mechanism controlling systemic inflammation and can be recruited by vagal or non-vagal afferent inputs (Martelli et al. 2014, 2019; Martelli et al., 2016; Komegae et al., 2018; McKinley et al., 2022). The carotid body provides a plausible arterial afferent entry point into this network, capable of detecting circulating inflammatory signals and converting them into sympathetic immune regulation.

In controlled or transient inflammation, this pathway may protect the host by restraining excessive cytokine production while preserving cardiovascular and metabolic adaptation. In prolonged sepsis, however, persistent anti-inflammatory autonomic signaling could theoretically contribute to poorly coordinated immune suppression, particularly when it coexists with continued tissue inflammation. Septic immunosuppression may represent a maladaptive extension of compensatory mechanisms rather than a simple absence of inflammation (Hotchkiss et al., 2013; Hotchkiss et al., 2016; Wiersinga and van der Poll, 2022; Gao et al., 2025). The evidence does not establish that carotid body signaling causes sepsis-induced immunosuppression, but it identifies a testable mechanism linking arterial cytokine sensing to autonomic immune control.

### 4.4 Autonomic coordination of thermoregulation and metabolism

Carotid body input may also influence thermogenesis, adrenal activity, hepatic glucose production, substrate mobilization, and energy redistribution through brainstem and sympathetic pathways. These outputs are relevant because fever is energetically costly, whereas hypothermia may reflect either failure of thermogenesis and perfusion or a regulated hypometabolic strategy that reduces oxygen demand and cardiovascular workload (Romanovsky and Székely, 1998; Garami et al., 2018; Ganeshan et al., 2019; Singer, 2014; Fan and Gautron, 2026). A functioning interoceptive system would need to integrate respiratory and circulatory requirements with the energetic cost of immune defense. The carotid body may contribute to this integration, although direct evidence linking carotid body activity to septic thermal phenotypes is currently lacking.

Loss of autonomic flexibility is a recognized feature of severe sepsis. Reduced heart rate variability has been evaluated as a predictor of mortality and may reflect impaired coordination of sympathetic and parasympathetic regulation (de Castilho et al., 2018). Because the carotid body projects to brainstem nuclei involved in both autonomic branches, altered carotid body signaling could plausibly contribute to this phenotype. However, heart rate variability is affected by sedation, mechanical ventilation, vasopressor therapy, age, comorbidities, pain, fever, and disease severity. It should therefore be interpreted as evidence of autonomic dysregulation, not as a specific marker of carotid body dysfunction.

## 5 Inflammation-induced carotid body remodeling and loss of sensory fidelity

### 5.1 Lipopolysaccharide-induced carotid body inflammation

The carotid body should not be viewed as a passive or structurally stable sensor during systemic inflammation. Infectious and inflammatory challenges can modify both its structure and function, potentially altering its capacity to encode changes in the arterial milieu. In cats, intravenous lipopolysaccharide caused systemic manifestations compatible with septic shock, increased basal carotid chemosensory discharge, and reduced ventilatory and chemosensory responses to hypoxia and nicotine. These functional changes were accompanied by disorganization of glomic lobules, increased connective-tissue deposition, and inflammatory-cell accumulation, supporting the concept of lipopolysaccharide-induced carotid body inflammation, or glomitis (Fernández et al. 2008, 2011).

This combination of elevated basal activity and reduced stimulus-evoked responsiveness illustrates a potential loss of sensory fidelity. The sensor may continue to generate afferent traffic but become less able to discriminate additional changes in oxygen, pH, or other stimuli. In sepsis, such distortion could impair both respiratory compensation and autonomic regulation because central circuits would receive a signal that no longer accurately encodes the magnitude or type of the arterial disturbance.

### 5.2 Intracellular inflammatory signaling in the chemoreceptor pathway

Inflammatory signaling occurs directly within the carotid chemoreceptor pathway in rats with lipopolysaccharide-induced sepsis syndrome. The carotid body and associated sensory ganglia express Toll-like receptor 4 as well as TNF-α and its receptors. Lipopolysaccharide activates MyD88-dependent signaling, including NF-κB, p38 MAPK, and ERK, and increases TNF-α/TNF receptor expression in tyrosine hydroxylase-positive cells of the chemosensory pathway (Fernández et al., 2011). These findings indicate that pathogen-associated signals can act directly on carotid body cells and afferent structures. Consequently, the carotid body may be both a detector of inflammation and a target of inflammatory injury.

### 5.3 Cytokine-mediated changes in excitability and chemosensory gain

Cytokines can reprogram carotid body function by altering glomus-cell excitability, intracellular Ca^2+^ signaling, neurotransmitter secretion, and afferent discharge. IL-1β receptors are strongly expressed in rat glomus cells, and IL-1β inhibits outward potassium currents, increases intracellular Ca^2+^, and increases carotid sinus nerve firing (Wang et al., 2002; Shu et al., 2007; Iturriaga et al., 2022). IL-6 increases intracellular Ca^2+^ and catecholamine secretion, suggesting a direct effect on transmitter release and chemosensory gain (Fan et al., 2009). TNF-α and its receptors are present in the carotid body, and TNF-α can modulate chemosensory activity, particularly under hypoxic conditions (Fernández et al. 2008, 2011; Zapata et al., 2011; Iturriaga et al., 2022).

These actions may be adaptive when cytokine exposure is brief and the resulting afferent signal recruits appropriate respiratory or anti-inflammatory responses. During severe or persistent inflammation, however, continued cytokine signaling may alter sensory gain, saturate afferent output, or change the relative encoding of hypoxia, acidosis, lactate, and glucose. The same inflammatory mediator may therefore function first as a signal to be detected and later as a driver of sensory dysfunction.

### 5.4 Persistent remodeling and transition to maladaptive signaling

Evidence from developmental models demonstrates that inflammatory exposure can produce persistent changes in carotid body structure and function. In newborn rats, early postnatal lipopolysaccharide exposure increased inflammatory cytokine mRNA in the carotid body, reduced type II cell volume, increased dopamine content, attenuated hypoxic chemosensitivity of the carotid sinus nerve, and impaired ventilatory responses to changes in oxygen tension one week after the insult (Master et al., 2016). Although this model is developmental rather than septic, it shows that inflammation can leave lasting morphological and functional effects on the carotid chemoreceptor system.

In sepsis, inflammation-induced remodeling could represent a transition point between adaptive inflammatory defense and immunometabolic decompensation. Initially, sensing of cytokines, lactate, glucose, hypoxemia, and acidosis may help coordinate ventilation, vascular tone, maintenance of arterial pressure, metabolic redistribution, and anti-inflammatory autonomic pathways. If the carotid body becomes inflamed or structurally disorganized, its ability to integrate these signals may deteriorate. An inflamed or remodeled carotid body may exhibit elevated tonic afferent activity but reduced stimulus-evoked responsiveness, contributing to impaired ventilatory and cardiovascular compensation, dysautonomia, disrupted thermoregulation, metabolic dysregulation, immune suppression, bioenergetic failure, and multiple-organ dysfunction.

This model does not imply that the carotid body is the primary cause of septic decompensation. Rather, it proposes that dysfunction of an arterial sensory checkpoint could amplify a broader failure of homeostatic coordination. The key mechanistic question is therefore not simply whether the carotid body is activated during sepsis, but whether it continues to encode the internal milieu accurately as inflammation progresses.

## 6 Clinical implications

The proposed role of the carotid body in sepsis remains primarily mechanistic, and the currently available clinical variables provide only indirect information about carotid body function. Nevertheless, respiratory, cardiovascular, autonomic, thermal, metabolic, and immune abnormalities may help identify septic phenotypes in which altered carotid chemoreflex signaling should be investigated. These variables should not be interpreted as specific markers of carotid body dysfunction but may provide a framework for translational assessment and future therapeutic studies.

### 6.1 Respiratory assessment

Direct assessment of carotid body function is not routinely performed in septic patients. Translational studies could combine hypoxic ventilatory responsiveness or other measures of carotid chemoreflex sensitivity with arterial blood gases, pH, serum lactate, respiratory support requirements, and organ dysfunction scores. Emerging human data from post-infectious syndromes indicate that infection-related conditions can alter carotid chemoreflex sensitivity and contribute to abnormal ventilatory responses (El-Medany et al., 2024). Although these findings cannot be extrapolated directly to acute sepsis, they support the feasibility of studying peripheral chemoreflex function in clinically relevant infection-related states.

### 6.2 Cardiovascular and autonomic assessment

Reduced heart rate variability has been repeatedly described in sepsis and evaluated as a predictor of mortality, suggesting that loss of autonomic flexibility accompanies severe systemic inflammation (de Castilho et al., 2018). Future studies could examine heart rate variability together with arterial pressure, vasopressor requirements, perfusion indices, lactate kinetics, and chemoreflex sensitivity. However, these measures are influenced by sedation, mechanical ventilation, vasopressors, age, comorbidities, pain, fever, and disease severity. They should therefore be interpreted as markers of cardiovascular or autonomic dysregulation rather than as specific evidence of carotid body dysfunction.

### 6.3 Thermal, metabolic, and immune phenotypes

Body temperature is one of the most accessible clinical variables in sepsis. Hypothermia is consistently associated with greater disease severity and worse outcomes, whereas febrile patients often have lower mortality than normothermic or hypothermic patients (Kushimoto et al., 2013; Rumbus et al., 2017; Thomas-Rüddel et al., 2021). In humans, however, it remains difficult to distinguish regulated energy-conserving hypothermia from hypothermia caused by thermoregulatory, circulatory, endocrine, or bioenergetic failure. There is no direct evidence that septic hypothermia is mediated by carotid body dysfunction. Temperature should therefore be interpreted as a clinical marker of immunometabolic state, not as a direct measure of carotid body activity.

Lactate provides a complementary window into metabolic and circulatory stress. Current sepsis guidelines recommend measuring serum lactate and using elevated lactate levels to guide resuscitation, while emphasizing contextual interpretation (Evans et al., 2021; Garcia-Alvarez et al., 2014; Hernandez et al., 2019). Within the framework proposed here, lactate is relevant both as a severity marker and as a potential circulating signal capable of activating carotid body glomus cells. Whether persistent hyperlactatemia increases tonic carotid body drive while inflammation-induced loss of dynamic gain impairs lactate-evoked compensatory responses in septic patients remains unknown (Torres-Torrelo et al., 2021).

Immune monitoring may add a further dimension. Reduced monocyte HLA-DR is among the most widely studied biomarkers of sepsis-induced immunosuppression and is associated with secondary infection and prolonged immune dysfunction (Hotchkiss et al., 2013). Simultaneous assessment of temperature, lactate, chemoreflex sensitivity, heart rate variability, monocyte HLA-DR, and organ dysfunction could help determine whether respiratory, autonomic, metabolic, and immune failures cluster within a common phenotype. At present, however, hypothermia, hyperlactatemia, reduced heart rate variability, and immune suppression remain indirect indicators and cannot establish a causal role for the carotid body (Kushimoto et al., 2013; Rumbus et al., 2017; Thomas-Rüddel et al., 2021; Evans et al., 2021; Garcia-Alvarez et al., 2014; Hernandez et al., 2019; Hotchkiss et al., 2013; Liu et al., 2022; de Castilho et al., 2018).

### 6.4 Potential therapeutic implications of preserving carotid body function

No treatment has been validated in sepsis that specifically targets the carotid body or restores carotid chemosensory function. Current management should therefore focus on correcting the systemic disturbances that may alter carotid body signaling, including infection, hypoxemia, ventilatory and acid–base abnormalities, hypotension, impaired tissue perfusion, dysglycemia, and the underlying causes of hyperlactatemia (Evans et al., 2021; Garcia-Alvarez et al., 2014; Hernandez et al., 2019). Experimental findings also suggest that limiting inflammation-induced carotid body remodeling or selectively modulating the carotid body–NTS–RVLM–splanchnic sympathetic pathway may represent future therapeutic strategies. However, inhibition of TLR4-and cytokine-related signaling could compromise antimicrobial defense, whereas excessive suppression or activation of sympathetic pathways could worsen hypotension, organ hypoperfusion, or immunosuppression (Wang et al., 2002; Shu et al., 2007; Fan et al., 2009; Fernández et al., 2008; Fernández et al., 2011; Iturriaga et al., 2022; Katayama et al., 2022a, Katayama et al., 2022b; Rosin et al., 2026; Hotchkiss et al., 2013; Hotchkiss et al., 2016; Wiersinga and van der Poll, 2022). Thus, future approaches should aim to preserve carotid body sensory fidelity rather than simply stimulate or inhibit the organ.

## 7 Proposed biphasic model and testable predictions

We propose a biphasic, context-dependent model. First, during systemic inflammation, the carotid body is exposed to cytokines, lactate, changes in glucose availability, hypoxemia, changes in CO_2_ and pH, pathogen-associated inflammatory signals, and energetic stress. Second, when the challenge is transient or controlled, integrated carotid body afferent activity may support ventilation and acid-base compensation while recruiting sympathetic pathways that preserve arterial pressure, vascular tone, organ perfusion, metabolic redistribution, and anti-inflammatory control. Third, when inflammation becomes severe or prolonged, lipopolysaccharide and cytokine signaling may alter glomus-cell excitability, local structure, chemosensory gain, and afferent output. Finally, basal afferent activity may remain elevated while stimulus-evoked responsiveness and chemosensory dynamic range decline, contributing to impaired respiratory and cardiovascular compensation, dysautonomia, thermoregulatory instability, metabolic dysregulation, immune suppression, bioenergetic failure, and multiple-organ dysfunction.

This model generates specific experimental predictions. Time-resolved animal studies should measure carotid sinus nerve activity in response to hypoxia, acidosis, lactate, glucose, and cytokines at different stages of systemic inflammation while simultaneously assessing ventilation, arterial pressure, sympathetic activity, splanchnic nerve activity, tissue perfusion, temperature, lactate, and immune responses. Histological and molecular analyses should determine whether glomitis, activation of Toll-like receptor signaling, altered cytokine expression, changes in dopamine content, or alterations in type II cells are associated with loss of stimulus discrimination.

Clinical studies should test whether septic patients with hypothermia, persistent hyperlactatemia, reduced heart rate variability, vasopressor dependence, immune suppression, or respiratory dysfunction show altered carotid chemoreflex sensitivity. Longitudinal measurements are essential because the hypothesis predicts a transition from early stimulus-coupled activation to elevated tonic activity with reduced stimulus-evoked responsiveness rather than a single static abnormality. If confirmed, carotid body dysfunction would represent not only a consequence of systemic inflammation but also a contributor to the loss of coordination among respiratory, cardiovascular, autonomic, metabolic, and immune responses.

**Fig. 1:**
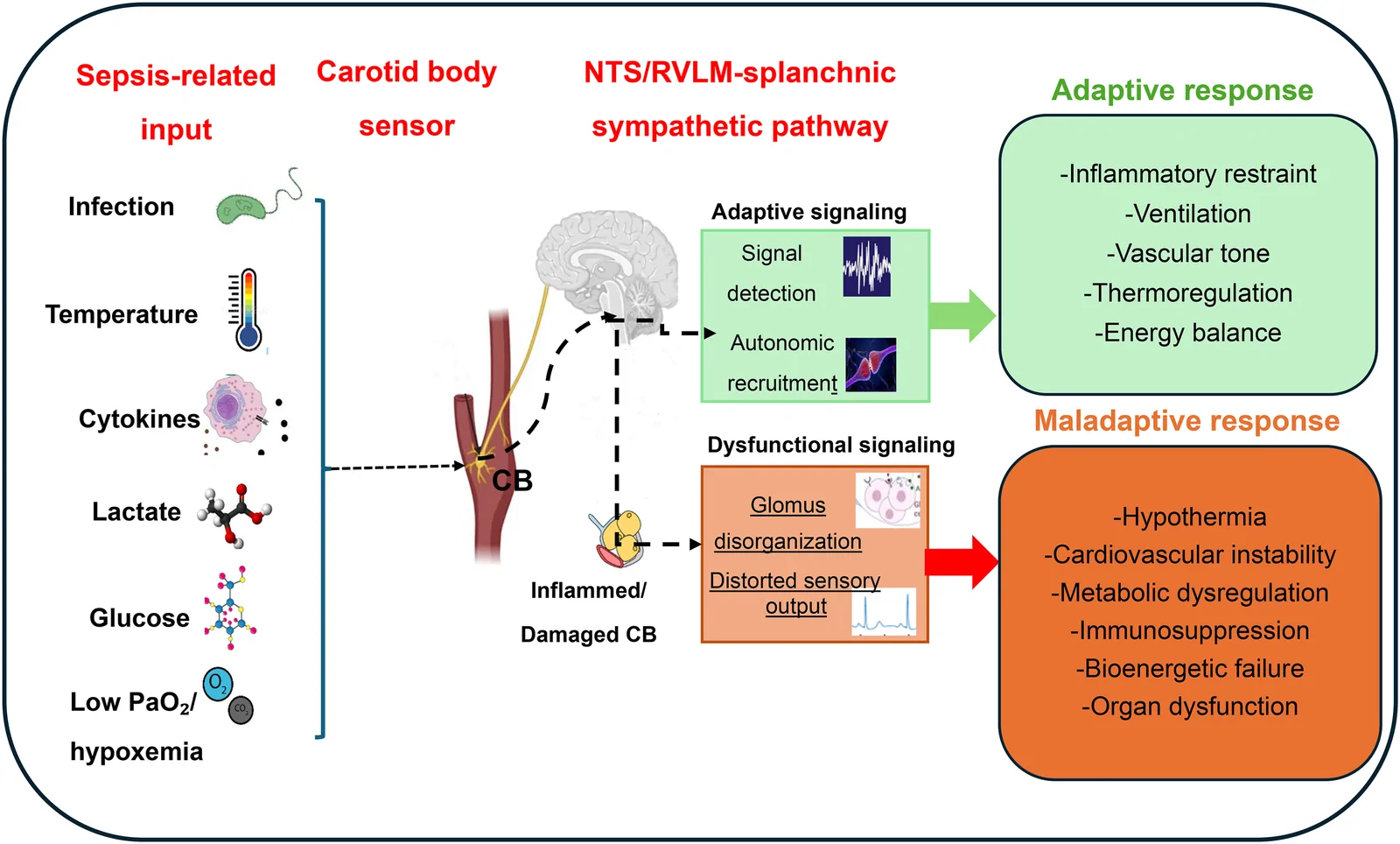
Proposed biphasic, context-dependent model of carotid body signaling during systemic inflammation and sepsis.

Within this framework, persistent hyperlactatemia is predicted initially to increase stimulus-coupled carotid body activation, but, during prolonged inflammation, to coexist with elevated tonic afferent discharge and reduced lactate- and hypoxia-evoked gain. Immunosuppression is predicted to be associated primarily with persistent tonic recruitment of the carotid body–splanchnic anti-inflammatory pathway rather than with complete loss of afferent activity. Hypothermia is predicted to accompany reduced stimulus-evoked sensory fidelity and impaired autonomic coordination, despite preserved or elevated basal afferent activity. These predictions refer to distinct components of carotid body output and can therefore be tested independently. The proposed biphasic and context-dependent model is illustrated in Fig. 1.

## 8 Conclusion

Sepsis represents a failure of coordinated homeostatic regulation involving immune, metabolic, respiratory, cardiovascular, thermal, endocrine, and autonomic systems. The carotid body is anatomically positioned to sample convergent arterial signals relevant to sepsis, including hypoxemia, changes in CO_2_ and pH, inflammatory cytokines, lactate, and potentially changes in glucose availability. Through its projections to brainstem autonomic circuits, carotid body signaling may contribute to the regulation of ventilation, hypoxic chemosensitivity, sympathetic activity, vascular tone, arterial pressure, tissue perfusion, and the splanchnic anti-inflammatory pathway. At the same time, the carotid body is a target of lipopolysaccharide- and cytokine-mediated inflammation. Structural and functional alterations induced by severe or persistent inflammation may reduce chemosensory dynamic range and impair the fidelity with which arterial disturbances are encoded. The carotid body should nevertheless be viewed as one component of a distributed interoceptive network rather than as an isolated sensor or the sole regulator of sepsis-related responses. Respiratory, cardiovascular, metabolic, and immune adaptations emerge from the integration of multiple peripheral and central sensory pathways. Carotid body dysfunction may therefore amplify the loss of homeostatic coordination by distorting arterial information conveyed to the brainstem, but it is unlikely to be the exclusive cause of autonomic or immunometabolic dysregulation. Direct clinical evidence remains limited. The central question for future studies is whether early stimulus-coupled carotid body activation gives way to elevated tonic activity with reduced stimulus-evoked responsiveness and impaired sensory discrimination, thereby contributing to cardiorespiratory, autonomic, and immune decompensation.

## CRediT authorship contribution statement

**Natalia C.S. Branco:** Conceptualization, Formal analysis, Validation, Writing – original draft, Writing – review & editing. **Isabela S. Ferracioli:** Formal analysis, Writing – original draft, Writing – review & editing. **Evelin C. Cárnio:** Conceptualization, Formal analysis, Supervision, Validation, Visualization, Writing – original draft, Writing – review & editing.

## Ethics approval and consent to participate

Not applicable. This is a review article and did not involve any new studies with human participants or animals performed by the authors.

## Consent for publication

Not applicable.

## Availability of data and materials

Not applicable. No original datasets were generated or analyzed for this review article.

## Declaration of generative AI and AI-assisted technologies in the manuscript preparation process

During the preparation of this work, the authors used ChatGPT, an AI-assisted technology developed by OpenAI, to support English-language refinement and improve readability and text organization. The authors reviewed, edited, and verified all AI-assisted outputs and take full responsibility for the accuracy, originality, and integrity of the manuscript.

## Funding

This work was supported by the 10.13039/501100001807São Paulo Research Foundation (FAPESP; grant 2024/16792-8 and fellowship 2026/07951-0 to I.S.F.), the 10.13039/501100003593National Council for Scientific and Technological Development (CNPq; ERC-CNPq 2025, grant 445287/2025-0), and the 10.13039/501100002322Coordination for the Improvement of Higher Education Personnel (CAPES; grant 88881.009149/2024-0).

## Declaration of competing interests

The authors declare that they have no known competing financial interests or personal relationships that could have appeared to influence the work reported in this paper. The authors have no employment, consultancies, stock ownership, honoraria, paid expert testimony, patent applications or registrations, grants or other funding, or affiliation with Brain, Behavior, & Immunity – Health as an Editor or Advisory Board Member to disclose. The authors have nothing to declare.

## Data Availability

No data was used for the research described in the article.

## References

[bib1] Basil M.C., Levy B.D. (2016). 'Specialized pro-resolving mediators: endogenous regulators of infection and inflammation'. Nat. Rev. Immunol..

[bib2] Bellavance M.A., Rivest S. (2014). The HPA - immune axis and the immunomodulatory actions of glucocorticoids in the brain. Front. Immunol..

[bib3] Borovikova L.V., Ivanova S., Zhang M., Yang H., Botchkina G.I., Watkins L.R., Wang H., Abumrad N., Eaton J.W., Tracey K.J. (2000). Vagus nerve stimulation attenuates the systemic inflammatory response to endotoxin. Nature.

[bib4] Brealey D., Singer M. (2003). Mitochondrial dysfunction in sepsis. Curr. Infect. Dis. Rep..

[bib5] Buckley C.D., Gilroy D.W., Serhan C.N. (2014). Proresolving lipid mediators and mechanisms in the resolution of acute inflammation. Immunity.

[bib6] Cárnio E.C., Branco L.G.S., Brognara F., Passaglia P., Foresti R., Motterlini R. (2025). Interplay between inflammatory reflex and energetic metabolism in endotoxaemia: a role for haem oxygenase 1?. Br. J. Pharmacol..

[bib7] Chrousos G.P. (1995). The hypothalamic-pituitary-adrenal axis and immune-mediated inflammation. N. Engl. J. Med..

[bib8] Conde S.V., Sacramento J.F., Guarino M.P. (2018). Carotid body: a metabolic sensor implicated in insulin resistance. Physiol. Genom..

[bib9] de Castilho F.M., Ribeiro A.L.P., Nobre V., Barros G., de Sousa M.R. (2018). 'Heart rate variability as predictor of mortality in sepsis: a systematic review'. PLoS One.

[bib10] El-Medany A., Adams Z.H., Blythe H.C., Hope K.A., Kendrick A.H., Abdala Sheikh A.P., Paton J.F.R., Nightingale A.K., Hart E.C. (2024). Carotid body dysregulation contributes to long COVID symptoms. Commun. Med..

[bib11] Evans L., Rhodes A., Alhazzani W., Antonelli M., Coopersmith C.M., French C., Machado F.R., Mcintyre L., Ostermann M., Prescott H.C., Schorr C., Simpson S., Wiersinga W.J., Alshamsi F., Angus D.C., Arabi Y., Azevedo L., Beale R., Beilman G., Belley-Cote E., Burry L., Cecconi M., Centofanti J., Coz Yataco A., De Waele J., Dellinger R.P., Doi K., Du B., Estenssoro E., Ferrer R., Gomersall C., Hodgson C., Møller M.H., Iwashyna T., Jacob S., Kleinpell R., Klompas M., Koh Y., Kumar A., Kwizera A., Lobo S., Masur H., McGloughlin S., Mehta S., Mehta Y., Mer M., Nunnally M., Oczkowski S., Osborn T., Papathanassoglou E., Perner A., Puskarich M., Roberts J., Schweickert W., Seckel M., Sevransky J., Sprung C.L., Welte T., Zimmerman J., Levy M. (2021). 'Surviving sepsis campaign: international guidelines for management of sepsis and septic shock 2021'. Intensive Care Med..

[bib12] Evans S.S., Repasky E.A., Fisher D.T. (2015). Fever and the thermal regulation of immunity: the immune system feels the heat. Nat. Rev. Immunol..

[bib13] Fan J., Zhang B., Shu H.F., Zhang X.Y., Wang X., Kuang F., Liu L., Peng Z.W., Wu R., Zhou Z., Wang B.R. (2009). Interleukin-6 increases intracellular Ca2+ concentration and induces catecholamine secretion in rat carotid body glomus cells. J. Neurosci. Res..

[bib14] Fan R., Gautron L. (2026). The poikilothermic hypothesis of sepsis. Crit. Care.

[bib15] Fernández R., González S., Rey S., Cortés P.P., Maisey K.R., Reyes E.P., Larraín C., Zapata P. (2008). Lipopolysaccharide-induced carotid body inflammation in cats: functional manifestations, histopathology and involvement of tumour necrosis factor-alpha. Exp. Physiol..

[bib16] Fernández R., Nardocci G., Simon F., Martin A., Becerra A., Rodríguez-Tirado C., Maisey K.R., Acuña-Castillo C., Cortes P.P. (2011). 'Lipopolysaccharide signaling in the carotid chemoreceptor pathway of rats with sepsis syndrome'. Respir. Physiol. Neurobiol..

[bib17] Ganeshan K., Nikkanen J., Man K., Leong Y.A., Sogawa Y., Maschek J.A., Van Ry T., Chagwedera D.N., Cox J.E., Chawla A. (2019). Energetic trade-offs and hypometabolic states promote disease tolerance. Cell.

[bib18] Gao L., Ortega-Sáenz P., García-Fernández M., González-Rodríguez P., Caballero-Eraso C., López-Barneo J. (2014). 'Glucose sensing by carotid body glomus cells: potential implications in disease'. Front. Physiol..

[bib19] Gao Q., Teng Y., Zhu L., Zhang W., Li Z. (2025). The immunosuppressive mechanisms induced by sepsis and the corresponding treatment strategies. Front. Immunol..

[bib20] Garami A., Steiner A.A., Romanovsky A.A. (2018). 'Fever and hypothermia in systemic inflammation'. Handb. Clin. Neurol..

[bib21] Garcia-Alvarez M., Marik P., Bellomo R. (2014). Sepsis-associated hyperlactatemia. Crit. Care.

[bib22] Gibbons T.D., Dempsey J.A., Thomas K.N., Ainslie P.N., Wilson L.C., Stothers T.A.M., Campbell H.A., Cotter J.D. (2022). Carotid body hyperexcitability underlies heat-induced hyperventilation in exercising humans. J. Appl. Physiol..

[bib23] Hernandez G., Bellomo R., Bakker J. (2019). The ten pitfalls of lactate clearance in sepsis. Intensive Care Med..

[bib24] Hotchkiss R.S., Moldawer L.L., Opal S.M., Reinhart K., Turnbull I.R., Vincent J.L. (2016). 'Sepsis and septic shock'. Nat. Rev. Dis. Primers.

[bib25] Hotchkiss R.S., Monneret G., Payen D. (2013). Sepsis-induced immunosuppression: from cellular dysfunctions to immunotherapy. Nat. Rev. Immunol..

[bib26] Iturriaga R. (2018). Translating carotid body function into clinical medicine. J. Physiol..

[bib27] Iturriaga R., Del Rio R., Alcayaga J. (2022). Carotid body inflammation: role in hypoxia and in the anti-inflammatory reflex. Physiology.

[bib28] Joyner M.J., Limberg J.K., Wehrwein E.A., Johnson B.D. (2018). 'Role of the carotid body chemoreceptors in glucose homeostasis and thermoregulation in humans'. J. Physiol..

[bib29] Katayama P.L., Leirão I.P., Kanashiro A., Luiz J.P.M., Cunha F.Q., Navegantes L.C.C., Menani J.V., Zoccal D.B., Colombari D.S.A., Colombari E. (2022). The carotid body detects circulating tumor necrosis factor-alpha to activate a sympathetic anti-inflammatory reflex. Brain Behav. Immun..

[bib30] Katayama P.L., Leirão I.P., Kanashiro A., Menani J.V., Zoccal D.B., Colombari D.S.A., Colombari E. (2022). The carotid body: a novel key player in neuroimmune interactions. Front. Immunol..

[bib31] Komegae E.N., Farmer D.G.S., Brooks V.L., McKinley M.J., McAllen R.M., Martelli D. (2018). 'Vagal afferent activation suppresses systemic inflammation via the splanchnic anti-inflammatory pathway'. Brain Behav. Immun..

[bib32] Kotas M.E., Medzhitov R. (2015). 'Homeostasis, inflammation, and disease susceptibility'. Cell.

[bib33] Kumar P., Prabhakar N.R. (2012). Peripheral chemoreceptors: function and plasticity of the carotid body. Compr. Physiol..

[bib34] Kushimoto S., Gando S., Saitoh D., Mayumi T., Ogura H., Fujishima S., Araki T., Ikeda H., Kotani J., Miki Y., Shiraishi S., Suzuki K., Suzuki Y., Takeyama N., Takuma K., Tsuruta R., Yamaguchi Y., Yamashita N., Aikawa N., JAAM Sepsis Registry Study Group (2013). The impact of body temperature abnormalities on the disease severity and outcome in patients with severe sepsis: an analysis from a multicenter, prospective survey of severe sepsis. Crit. Care.

[bib35] Liu D., Huang S.Y., Sun J.H., Zhang H.C., Cai Q.L., Gao C., Li L., Cao J., Xu F., Zhou Y., Guan C.X., Jin S.W., Deng J., Fang X.M., Jiang J.X., Zeng L. (2022). 'Sepsis-induced immunosuppression: mechanisms, diagnosis and current treatment options'. Milit. Med. Res..

[bib36] Liu S., Yang T., Jiang Q., Zhang L., Shi X., Liu X., Li X. (2024). 'Lactate and Lactylation in Sepsis: a Comprehensive Review'. J. Inflamm. Res..

[bib38] Martelli D., Farmer D.G., Yao S.T. (2016). The splanchnic anti-inflammatory pathway: could it be the efferent arm of the inflammatory reflex?. Exp. Physiol..

[bib37] Martelli D., Farmer D.G.S., McKinley M.J., Yao S.T., McAllen R.M. (2019). Anti-inflammatory reflex action of splanchnic sympathetic nerves is distributed across abdominal organs. Am. J. Physiol. Regul. Integr. Comp. Physiol..

[bib39] Martelli D., Yao S.T., Mancera J., McKinley M.J., McAllen R.M. (2014). 'Reflex control of inflammation by the splanchnic anti-inflammatory pathway is sustained and independent of anesthesia'. Am. J. Physiol. Regul. Integr. Comp. Physiol..

[bib40] Master Z.R., Porzionato A., Kesavan K., Mason A., Chavez-Valdez R., Shirahata M., Gauda E.B. (2016). 'Lipopolysaccharide exposure during the early postnatal period adversely affects the structure and function of the developing rat carotid body'. J. Appl. Physiol..

[bib41] McKinley M.J., Martelli D., Trevizan-Baú P., McAllen R.M. (2022). Divergent splanchnic sympathetic efferent nerve pathways regulate interleukin-10 and tumour necrosis factor-α responses to endotoxaemia. J. Physiol..

[bib42] Medzhitov R. (2008). 'Origin and physiological roles of inflammation'. Nature.

[bib43] Nathan C. (2002). Points of control in inflammation. Nature.

[bib44] Pavlov V.A., Tracey K.J. (2012). The vagus nerve and the inflammatory reflex--linking immunity and metabolism. Nat. Rev. Endocrinol..

[bib45] Protti A., Singer M. (2006). Bench-to-bedside review: potential strategies to protect or reverse mitochondrial dysfunction in sepsis-induced organ failure. Crit. Care.

[bib46] Romanovsky A.A., Székely M. (1998). 'Fever and hypothermia: two adaptive thermoregulatory responses to systemic inflammation'. Med. Hypotheses.

[bib47] Rosas-Ballina M., Olofsson P.S., Ochani M., Valdés-Ferrer S.I., Levine Y.A., Reardon C., Tusche M.W., Pavlov V.A., Andersson U., Chavan S., Mak T.W., Tracey K.J. (2011). Acetylcholine-synthesizing T cells relay neural signals in a vagus nerve circuit. Science.

[bib48] Rosin T.F.V., Corrêa L.E.C., Morais R.S., Leirão I.P., Cardoso L.M., Medeiros F.C., Lopes L.S., Colombari D.S.A., Colombari E., Basso F.G., Zoccal D.B., Katayama P.L. (2026). Carotid body sensing of systemic inflammation contributes to recruitment of the splanchnic anti-inflammatory pathway. Brain Behav. Immun..

[bib49] Rumbus Z., Matics R., Hegyi P., Zsiboras C., Szabo I., Illes A., Petervari E., Balasko M., Marta K., Miko A., Parniczky A., Tenk J., Rostas I., Solymar M., Garami A. (2017). 'Fever Is Associated with Reduced, Hypothermia with Increased Mortality in Septic Patients: a Meta-Analysis of Clinical Trials'. PLoS One.

[bib50] Shu H.F., Wang B.R., Wang S.R., Yao W., Huang H.P., Zhou Z., Wang X., Fan J., Wang T., Ju G. (2007). 'IL-1beta inhibits IK and increases [Ca2+]i in the carotid body glomus cells and increases carotid sinus nerve firings in the rat'. Eur. J. Neurosci..

[bib51] Singer M. (2014). The role of mitochondrial dysfunction in sepsis-induced multi-organ failure. Virulence.

[bib52] Singer M., Deutschman C.S., Seymour C.W., Shankar-Hari M., Annane D., Bauer M., Bellomo R., Bernard G.R., Chiche J.D., Coopersmith C.M., Hotchkiss R.S., Levy M.M., Marshall J.C., Martin G.S., Opal S.M., Rubenfeld G.D., van der Poll T., Vincent J.L., Angus D.C. (2016). The third international consensus definitions for sepsis and septic shock (Sepsis-3). JAMA.

[bib53] Thomas-Rüddel D.O., Hoffmann P., Schwarzkopf D., Scheer C., Bach F., Komann M., Gerlach H., Weiss M., Lindner M., Rüddel H., Simon P., Kuhn S.O., Wetzker R., Bauer M., Reinhart K., Bloos F., MEDUSA study group (2021). Fever and hypothermia represent two populations of sepsis patients and are associated with outside temperature. Crit. Care.

[bib54] Torres-Torrelo H., Ortega-Sáenz P., Gao L., López-Barneo J. (2021). Lactate sensing mechanisms in arterial chemoreceptor cells. Nat. Commun..

[bib55] Tracey K.J. (2002). The inflammatory reflex. Nature.

[bib56] van der Poll T., van de Veerdonk F.L., Scicluna B.P., Netea M.G. (2017). The immunopathology of sepsis and potential therapeutic targets. Nat. Rev. Immunol..

[bib57] Vandewalle J., Libert C. (2020). Glucocorticoids in sepsis: to be or not to be. Front. Immunol..

[bib58] Wang H., Yu M., Ochani M., Amella C.A., Tanovic M., Susarla S., Li J.H., Yang H., Ulloa L., Al-Abed Y., Czura C.J., Tracey K.J. (2003). 'Nicotinic acetylcholine receptor alpha7 subunit is an essential regulator of inflammation'. Nature.

[bib59] Wang X., Wang B.R., Duan X.L., Zhang P., Ding Y.Q., Jia Y., Jiao X.Y., Ju G. (2002). Strong expression of interleukin-1 receptor type I in the rat carotid body. J. Histochem. Cytochem..

[bib60] Wiersinga W.J., van der Poll T. (2022). Immunopathophysiology of human sepsis. EBioMedicine.

[bib61] Wrotek S., LeGrand E.K., Dzialuk A., Alcock J. (2021). Let fever do its job: the meaning of fever in the pandemic era. Evol. Med. Public Health.

[bib62] Zapata P., Larraín C., Reyes P., Fernández R. (2011). Immunosensory signalling by carotid body chemoreceptors. Respir. Physiol. Neurobiol..

[bib63] Zhang D., Tang Z., Huang H., Zhou G., Cui C., Weng Y., Liu W., Kim S., Lee S., Perez-Neut M., Ding J., Czyz D., Hu R., Ye Z., He M., Zheng Y.G., Shuman H.A., Dai L., Ren B., Roeder R.G., Becker L., Zhao Y. (2019). 'Metabolic regulation of gene expression by histone lactylation'. Nature.

[bib64] Zhang T., Chen L., Kueth G., Shao E., Wang X., Ha T., Williams D.L., Li C., Fan M., Yang K. (2024). Lactate's impact on immune cells in sepsis: unraveling the complex interplay. Front. Immunol..

[bib65] Zhang W., Fang X., Gao C., Song C., He Y., Zhou T., Yang X., Shang Y., Xu J. (2023). MDSCs in sepsis-induced immunosuppression and its potential therapeutic targets. Cytokine Growth Factor Rev..

